# Guardians of consent: enhancing rape prevention through social control mechanisms

**DOI:** 10.3389/fsoc.2024.1487451

**Published:** 2024-12-13

**Authors:** Sudip Bhattacharya, Amarjeet Singh

**Affiliations:** ^1^Department of Community and Family Medicine, All India Institute of Medical Sciences, Deoghar (AIIMS Deoghar), Jharkhand, India; ^2^Department of Community Medicine, Post Graduate Institute of Medical Education and Research (PGIMER), Chandigarh, India

**Keywords:** sexual offence, rape, victim, social control, rape prevention and control

## Abstract

**Introduction:**

Rape is a severe violation involving non-consensual sexual acts, often accompanied by force, threats, or coercion, leading to profound physical, emotional, and social consequences for survivors.

**Aim:**

This review aims to examine and synthesize research on effective strategies for rape prevention and intervention, with a particular focus on social control mechanisms, legal frameworks, cultural change, educational programs, public awareness campaigns, community vigilance, victim support services, and the influence of digital media.

**Methodology:**

A comprehensive review was conducted by searching PubMed, Scopus, PsycINFO, and Google Scholar for peer-reviewed articles, policy papers, and reports from the past 20 years. Keywords like “rape prevention,” “legal frameworks,” and “consent education” were used with Boolean operators to refine the focus. Articles addressing prevention strategies, social control, legal reforms, or public awareness with empirical findings or theoretical insights were included, while unrelated studies were excluded. From 6500 records, 39 studies were ultimately included after screening and eligibility assessments.

**Results:**

The global and national burden of rape is substantial, with significant prevalence differences influenced by cultural, socio-economic, and legal factors. Effective prevention of rape necessitates a multifaceted approach that includes stringent legal frameworks, comprehensive education on consent, public awareness campaigns, social control, and community vigilance.

**Discussion:**

Social control plays a critical role in these prevention strategies, encompassing both formal mechanisms like legal sanctions and informal controls such as societal norms and cultural attitudes. Challenges to these efforts include persistent rape myths, victim-blaming, inconsistent legal definitions of consent, and the evolving complexities of digital media. However, global examples such as Sweden’s consent-based legal reforms and the UK’s cultural shift through public campaigns demonstrate that these challenges can be addressed effectively. National efforts, including the “It’s On Us” campaign in the United States and legal reforms in India, further highlight the importance of tailored interventions to address specific contextual challenges.

**Conclusion:**

Ultimately, overcoming these challenges requires an integrated strategy that combines legal reforms, educational initiatives, cultural change, and robust support systems for survivors. By learning from successful global and national models, societies can build more effective frameworks for preventing rape and ensuring justice for those affected.

## Introduction

Rape is a profound violation involving non-consensual sexual intercourse or activity, where the victim is subjected to force, threats, or coercion. This form of sexual violence transcends physical assault, impacting survivors’ mental, emotional, and social well-being. It can occur in various contexts, including intimate relationships, and can be perpetrated by strangers or acquaintances. The trauma inflicted by rape is severe and pervasive, leading to long-term consequences for victims, including psychological disorders such as post-traumatic stress disorder (PTSD), depression, anxiety, and chronic physical health issues. Additionally, rape often results in significant social stigma and isolation, further compounding the challenges faced by survivors (Kalra and Bhugra, 2013).

### Burden of rape

The global burden of rape is substantial and deeply concerning. According to the World Health Organization (WHO), intimate partner violence, which frequently includes rape, affects about 30% of women worldwide (Violence Against Women, 2013). The WHO’s Global Status Report on Violence Prevention highlights that sexual violence is a major public health issue affecting millions of individuals each year, with significant differences in prevalence depending on regional and cultural factors. For example, in regions like Sub-Saharan Africa, the rates of sexual violence are particularly high due to factors such as gender inequality, conflict, and poverty (UN Women–Headquarters, 2024). The Global Burden of Disease Study underscores that sexual violence, including rape, contributes to a considerable global health burden, impacting not only survivors but also resulting in substantial economic costs associated with healthcare, legal proceedings, and lost productivity (Brown et al., 2019).

Research indicates that rape and sexual violence are often underreported due to various barriers, including fear of stigma, mistrust of the justice system, and concerns about victim-blaming. The prevalence of rape in conflict zones is also alarmingly high, with sexual violence being used as a tool of war. Reports from organizations such as Amnesty International and Human Rights Watch highlight the widespread use of rape in conflicts in countries like the Democratic Republic of the Congo and Syria, where it is employed systematically to terrorize and control populations (Meetings Coverage and Press Releases, 2023). This global epidemic necessitates a coordinated international response to address both the immediate and long-term needs of survivors and to tackle the root causes of sexual violence.

In the United States, the National Sexual Violence Resource Center reports that approximately 1 in 5 women and 1 in 71 men have experienced rape at some point in their lives (National Sexual Violence Resource Center, 2010). The National Crime Victimization Survey conducted by the U.S. Department of Justice reveals that a significant number of rapes are not reported to authorities, with underreporting due to fear of not being believed, the stigma attached to rape, and a lack of faith in the justice system. This discrepancy highlights the need for improved support systems and reforms in the legal system to better address the needs of survivors and encourage reporting (Ballard Brief, 2023).

In the United Kingdom, the Office for National Statistics estimates that about 1 in 6 women and 1 in 33 men have been victims of sexual assault or rape since the age of 16 (Office for National Statistics, 2013). The Crime Survey for England and Wales provides data that reveal high levels of underreporting and significant gaps in the effectiveness of the justice system in prosecuting rape cases. Despite various initiatives and improvements in legal frameworks, challenges such as inadequate forensic evidence collection and victim-blaming attitudes persist, affecting the ability to achieve justice for survivors (Office for National Statistics, 2013).

In India, the situation is similarly dire, with a high prevalence of rape and sexual violence, often exacerbated by deeply entrenched patriarchal norms and societal attitudes that blame victims and minimize the severity of these crimes (Himabindu et al., 2014). Studies indicate that despite legislative changes and increased awareness campaigns, cultural barriers and systemic failures continue to hinder efforts to prevent rape and provide justice for survivors (Dworkin and Weaver, 2021). Initiatives by NGOs and advocacy groups are crucial in challenging these norms, promoting gender equality, and supporting survivors in navigating the legal system.

### Aim

This systemic review aims to examine and synthesize research on effective strategies for rape prevention and intervention, with a particular focus on social control mechanisms, legal frameworks, cultural change, educational programs, public awareness campaigns, community vigilance, victim support services, and the influence of digital media. As prevention of rape needs multipronged approach.

## Methodology

### Search strategy

To ensure comprehensive coverage, the literature search included articles from multidisciplinary databases such as PubMed, Scopus, PsycINFO, and Google Scholar. The search targeted peer-reviewed articles, policy papers, and reports from the past 20 years, reflecting recent advances in this area.

### Keywords

Terms such as “rape prevention,” “sexual violence prevention,” “legal frameworks for rape,” “consent education,” “rape myths,” “rape social control,” “rape survivor support,” and “rape prevention campaigns” were combined using Boolean operators. Terms like “global rape prevention” or specific campaigns (e.g., “It’s On Us”) refined the scope.

### Inclusion criteria

Articles were included if they (a) addressed rape prevention strategies or interventions, (b) discussed the role of social control, legal reforms, or public awareness, and (c) presented empirical findings or substantial theoretical insights.

### Exclusion criteria

Articles that do not directly address rape prevention or focus exclusively on outcomes unrelated to prevention was excluded.

### Search results

**Figure 1:**
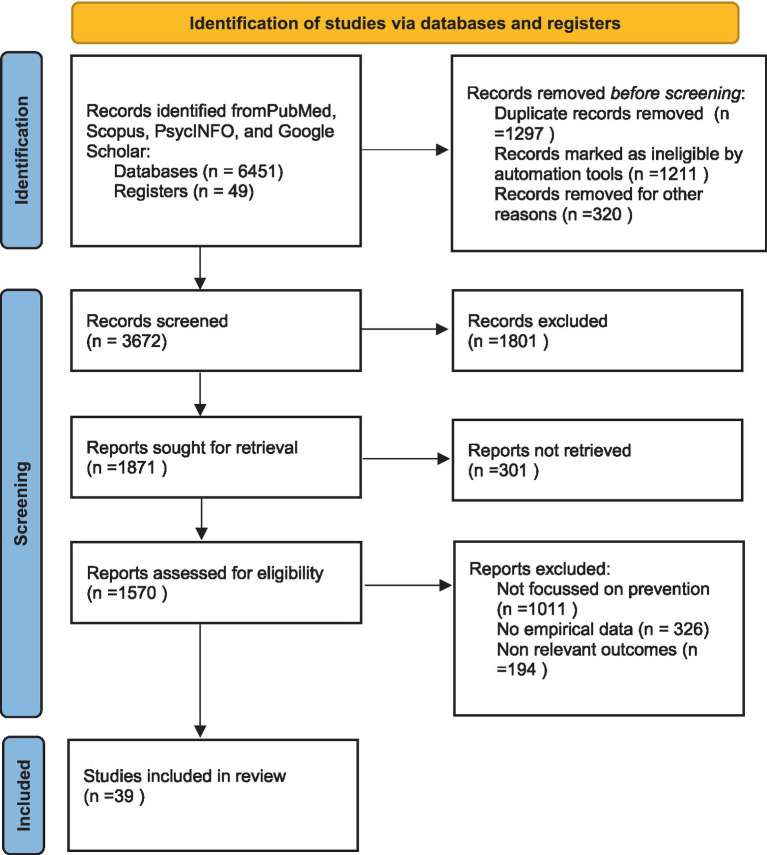
Search strategies for inclusion of studies.

Out of 6500 records, 2828 records were removed before screened due to duplication, ineligibility and other reasons. Total 3672 records were screened and later 1801 were excluded. In the next step 1871 reports sought for retrieval among them 301 records were not retrieved. Total 1570 reports were assessed for eligibility and later 1531 reports were excluded due to lack of empirical data, lack of focus on prevention and non-relevant outcomes. So we included 39 studies for this review (Figure 1).

## Results and discussion

We have conducted this review which was aimed to examine and synthesize research on effective strategies for rape prevention and intervention, with a particular focus on social control mechanisms, legal frameworks, cultural change, educational programs, public awareness campaigns, community vigilance, victim support services, and the influence of digital media. The theme wise findings are presented in the sections below.

### Social control

Social control is a fundamental concept in sociology that refers to the various methods and processes through which societies regulate individual and group behavior, thereby maintaining social order and ensuring conformity to established norms. These mechanisms can be categorized into formal and informal controls, each playing a distinct yet complementary role in shaping how people behave and interact within a community (Benson, 1989).

Formal social control involves the creation and enforcement of rules and regulations by authoritative institutions such as governments, courts, and law enforcement agencies. These institutions establish laws and policies that clearly define acceptable and unacceptable behaviors, along with corresponding sanctions for violations. For example, laws against theft, assault, and other crimes are enforced by police and judicial systems, which impose penalties like fines, imprisonment, or community service on those who break these laws. The presence of such formal controls is crucial for maintaining order, as they provide a clear and structured framework for behavior, ensuring that individuals are aware of the consequences of deviance (Benson, 1989).

Informal social control, on the other hand, operates through less explicit but equally powerful means, relying on the influence of social norms, values, customs, and expectations that are learned and internalized over time. This type of control is exerted through the socialization process, beginning in childhood and continuing throughout life, as individuals interact with family members, peers, educators, and other community members. For instance, a person may conform to societal norms regarding politeness, honesty, or modesty not because of the fear of legal punishment, but because of the desire to be accepted and respected within their social circle. Informal social control is often enforced through social approval or disapproval, such as praise, criticism, gossip, or ostracism, which can significantly influence behavior by rewarding conformity and discouraging deviance (Benson, 1989).

The primary purpose of social control, whether formal or informal, is to maintain order within society, promote stability, and protect the rights and safety of individuals. By guiding behavior and encouraging conformity to societal expectations, social control helps prevent chaos and conflict, allowing society to function smoothly. For example, formal controls like traffic laws help prevent accidents and ensure that roadways are safe for all users, while informal controls like social norms around honesty and fairness help maintain trust and cooperation in relationships and business dealings (Benson, 1989).

Furthermore, social control plays a critical role in supporting social institutions and relationships that are essential for the cohesion and continuity of society. Families, schools, religious institutions, and workplaces all rely on social control to enforce norms and expectations that enable them to function effectively. For example, schools use both formal rules (like codes of conduct) and informal expectations (like respect for teachers) to create a learning environment conducive to education. Similarly, workplaces enforce rules and norms that ensure productivity and professionalism, contributing to the overall stability and economic health of society (Benson, 1989).

Preventing rape through social control is a complex and ongoing process that requires a comprehensive approach, integrating legal, educational, social, and cultural strategies. These efforts must be synchronized and sustained to create an environment where rape is not only condemned but also significantly less likely to occur. Each component of this approach plays a distinct yet interconnected role in shaping a society that actively works to prevent sexual violence (Bonar et al., 2022; DeGue et al., 2014; Gaffney et al., 2021; O’Doherty et al., 2023).

### Strict legal frameworks

A cornerstone of preventing rape through social control is the establishment of stringent legal frameworks that clearly define and penalize sexual violence. Sweden serves as a leading example with its progressive laws that criminalize any sexual act without explicit consent, known as the “consent-based” approach to sexual offenses. This legal standard shifts the focus from the actions of the victim to the actions of the perpetrator, ensuring that the absence of consent is the central criterion in prosecuting rape. Such laws not only provide justice for survivors but also act as a powerful deterrent to potential offenders. By establishing clear legal consequences for sexual violence, these frameworks send a strong societal message that rape will not be tolerated and that perpetrators will be held accountable (Cedeno and Bohlen, 2024).

### Educational programs

Education plays a critical role in preventing rape by addressing the root causes of sexual violence, such as ignorance about consent, gender inequality, and toxic masculinity. Programs like “It’s On Us” in the United States are designed to educate individuals, particularly young people, about the importance of consent and respect in all relationships (Brady et al., 2022). These programs often include workshops, seminars, and campaigns that challenge harmful stereotypes and promote healthy, respectful interactions. By instilling these values early on, educational initiatives can reshape attitudes towards sexual violence, reducing the likelihood of rape by fostering a culture of mutual respect and understanding. Furthermore, education can empower bystanders to intervene in situations that may lead to sexual violence, creating a community-wide commitment to preventing rape.

### Public awareness campaigns

Public awareness campaigns are essential in shifting societal perceptions and attitudes towards rape. Initiatives like the “No Means No” campaign have been instrumental in challenging pervasive rape myths and victim-blaming narratives that have historically protected perpetrators and silenced survivors (Willis and Marcantonio, 2023). These campaigns use media, public service announcements, and social media platforms to spread messages that emphasize the importance of consent and the need to hold rapists accountable for their actions. By reaching a wide audience, public awareness efforts help to dismantle the cultural acceptance of rape, replacing it with a strong societal condemnation of sexual violence. This change in public perception is crucial for creating an environment where survivors feel supported in coming forward, and where the community as a whole is committed to preventing rape.

### Community vigilance

Community-based programs are another vital component of social control in preventing rape. Initiatives such as neighborhood watch programs enhance community vigilance by encouraging residents to look out for one another and report suspicious behavior (Brown et al., 2019). These programs create a sense of collective responsibility, where potential offenders are deterred by the knowledge that their actions are being monitored by the community. Additionally, community programs can foster a supportive environment for survivors by providing resources, such as hotlines and support groups, where they can seek help and report crimes without fear of judgment or retaliation. The active involvement of the community in preventing rape not only increases the likelihood of deterring sexual violence but also strengthens the social fabric by promoting solidarity and mutual care (Campbell et al., 1999).

Bystander intervention plays a critical role in rape prevention by encouraging individuals within a community to recognize and act against potential or occurring instances of sexual violence. This approach empowers bystanders—often peers, friends, or colleagues—to intervene safely, whether through distraction, direct action, or seeking assistance, which can prevent assault or support the victim. The alignment of bystander intervention with social control theory is evident, as social control theory posits that individuals’ behaviors are influenced and regulated by societal norms, relationships, and communal values. Bystander programs leverage this concept by fostering a culture where individuals feel both responsible for and capable of acting against harmful behaviors, reinforcing positive social norms and reducing acceptance of rape myths and victim-blaming attitudes. In practice, bystander intervention training encourages community members to internalize anti-violence values, making the prevention of rape a shared responsibility, thereby strengthening informal social controls that inhibit such behavior. Thus, bystander intervention not only prevents potential incidents but also fosters a culture of accountability and respect, vital to sustaining a community committed to preventing sexual violence (Leone et al., 2017).

Examples of bystander intervention programs illustrate how this approach effectively contributes to rape prevention by promoting active community involvement and reinforcing positive social norms. One prominent example is the Green Dot program, which teaches community members strategies to safely intervene in situations with a risk of sexual violence. Green Dot, implemented in schools and universities, encourages participants to use methods like distraction, delegation, and direct intervention, aligning with social control theory by fostering an environment where peers feel accountable for each other’s safety. Research has shown that Green Dot participants are more likely to intervene in potentially harmful situations, thereby reducing violence acceptance and fostering safer community norms (Davidov et al., 2019).

Another example is the Bringing in the Bystander program, which educates individuals on recognizing and responding to high-risk situations for sexual assault. This program not only provides practical intervention skills but also emphasizes the social responsibility of the bystander, reinforcing social control theory by making community norms a pivotal part of preventing rape. Participants of Bringing in the Bystander report increased confidence and willingness to intervene, showing that when social controls are strengthened through bystander education, community members are more likely to act in accordance with anti-violence norms (Inman et al., 2018).

### Support services for survivors

Support services for survivors of rape are crucial in both helping individuals heal and in preventing further violence. Organizations like the Rape, Abuse & Incest National Network (RAINN) in the United States provide critical resources, including counseling, legal assistance, and advocacy, to survivors of sexual violence (Meetings Coverage and Press Releases, 2023). These services are essential for encouraging survivors to report their assaults, which increases the chances of prosecuting offenders and preventing them from committing further crimes. Moreover, support services help to combat the stigma often associated with rape, empowering survivors to speak out and seek justice. By providing a safety net for survivors, these services play a key role in the broader strategy of preventing rape, ensuring that survivors are not left to suffer in silence and those perpetrators are held accountable (Banvard-Fox et al., 2020).

### Cultural change

Finally, a long-term solution to preventing rape lies in challenging and changing the cultural norms that perpetuate gender inequality and sexual violence. In many parts of the world, including India, NGOs have been working tirelessly to dismantle patriarchal attitudes that view women as subordinate to men, which can contribute to the normalization of sexual violence (Kalra and Bhugra, 2013). These organizations promote gender equality through education, advocacy, and community engagement, aiming to transform societal values that support or excuse rape. Cultural change is a slow and often challenging process, but it is essential for creating a society where sexual violence is not tolerated and where all individuals, regardless of gender, are treated with respect and dignity.

### Challenges in social control for preventing rape

Addressing the prevention of rape through social control is fraught with significant challenges that arise from cultural norms, legal inconsistencies, and the influence of digital media. One of the most pervasive obstacles is the persistence of rape myths and victim-blaming attitudes, which undermine efforts to hold perpetrators accountable and support survivors. Rape myths, such as the belief that victims are responsible for the assault due to their behavior, clothing, or prior relationships, can severely impact the reporting and prosecution of sexual violence. For example, research highlights that in various cultures, patriarchal norms contribute to a widespread belief that men have a right to women’s bodies, reinforcing a climate where rape is tolerated or even justified. These myths not only discourage victims from seeking help due to fear of judgment or disbelief but also create leniency toward offenders by shifting the focus away from the perpetrator’s actions (Bhattacharya and Singh, 2017).

In addition to cultural challenges, inconsistencies in legal frameworks and their enforcement pose significant barriers to effectively preventing rape (National Academies of Sciences, Engineering, and Medicine et al., 2018a). Laws regarding sexual violence vary widely across jurisdictions, with some regions having outdated or inadequate legal definitions of consent and limited recognition of various forms of sexual violence, including marital rape. Even in countries with comprehensive legal protections, systemic issues such as corruption, insufficient law enforcement training, and social biases can impede the effective implementation of these laws. Survivors often face skepticism and hostility from authorities, further complicating their pursuit of justice (Lorenz et al., 2019). This inconsistency weakens the deterrent effect of legal sanctions and creates barriers for survivors seeking redress, highlighting the need for legal reform and better enforcement mechanisms.

Informal mechanisms of social control also face considerable challenges in preventing rape. Cultural and religious beliefs, along with entrenched traditional gender roles, often reinforce power imbalances that contribute to sexual violence (Bhattacharya and Singh, 2017). In many communities, discussions about sex and consent are taboo, which limits opportunities for education and open dialogue that could help prevent rape. Social stigma associated with sexual violence exacerbates these issues, leading to the isolation and ostracization of survivors. This stigma not only affects individuals but also perpetuates a broader culture of silence and denial that allows rape to continue unchecked (Lorenz et al., 2019). Challenging these deeply ingrained beliefs and promoting open conversations about consent and respect are crucial steps in addressing the informal social control mechanisms that contribute to the persistence of rape.

The advent of digital media and technology introduces additional complexities to the prevention of rape. While online platforms can be instrumental in raising awareness and providing support, they also pose challenges by facilitating the spread of rape culture through the sharing of non-consensual images, cyber bullying, and online harassment. The anonymity and vast reach of the internet complicate efforts to regulate and address these issues effectively. Existing legal frameworks often struggle to keep pace with the rapid evolution of technology, creating gaps in the regulation of online behavior (Marchant et al., 2011). This digital dimension requires innovative strategies and approaches to ensure that technology is used to support rather than undermine efforts to prevent rape and protect survivors.

### Examples of overcoming challenges in rape prevention

Globally, various initiatives illustrate effective strategies to overcome challenges in preventing rape. In Sweden, a pioneering approach to legal reform and education has made significant strides in addressing sexual violence. Sweden’s laws are among the most progressive in the world, with a clear and expansive definition of consent that requires explicit agreement for sexual activity (Tirado et al., 2023). This legal framework, combined with robust public education campaigns like the “Yes Means Yes” initiative, helps to reshape societal attitudes towards sexual consent and responsibility. It is also considered important to include recent findings on consent, which specify that sometimes ‘just yes is yes’ is not enough. In a study conducted by Duque E et al. highlights the significant impact of peer influence on sexual and affective relationships among teenagers and young adults, emphasizing how coercive discourse can lead to unwanted and violent relationships. Studies indicate that this coercive discourse, which pressures individuals to maintain toxic relationships, promotes an attraction to violence and can have adverse effects on health and well-being. The study underscores the importance of understanding consent within these dynamics, particularly as young women’s early experiences shape their future relationships. It critiques the prevailing notion of consent, which is often influenced by societal expectations and peer pressure, leading to situations where women feel obligated to comply with unwanted sexual practices. Research demonstrates that women frequently navigate coercive environments where their lack of consent is rationalized or ignored. The study also points out the complexities of consent in both stable and casual relationships, where communication about consent is often inadequate. Ultimately, the study calls for further investigation into how coercive discourse and the socialization of consent affect young women’s relationship trajectories, emphasizing the need for preventive socialization strategies from families, educational institutions, and peer groups to foster healthier relationships (Duque et al., 2024).

Sweden also invests heavily in training law enforcement and judicial personnel to handle sexual violence cases sensitively, ensuring that survivors receive the support they need and that perpetrators are held accountable (Flecha et al., 2020). Additionally, Sweden’s comprehensive approach includes support services for survivors, such as counseling and legal aid, which contribute to a high reporting rate and effective prosecution of sexual offenses (Tirado et al., 2023).

In the United Kingdom, efforts to address the informal mechanisms of social control and cultural attitudes towards sexual violence are exemplified by the “This Is Not an Invitation to Rape Me” campaign (Collaton et al., 2024). This initiative challenges rape myths and victim-blaming attitudes by promoting a clear message about consent and respect. The campaign, supported by public figures and media outreach, aims to shift cultural norms and reduce the stigma associated with sexual violence. Moreover, the UK’s recent legislative reforms, including the introduction of the Domestic Abuse Act 2021, which recognizes and addresses various forms of abuse, reflect a broader commitment to improving legal protections for survivors and enhancing support services (Aldridge, 2021). These measures, combined with community engagement and public awareness efforts, demonstrate a comprehensive approach to tackling rape and supporting survivors.

In the United States, the “It’s On Us” campaign represents a significant national effort to overcome cultural barriers and promote a culture of consent. Launched by the Obama Administration in 2014, this campaign aims to raise awareness about sexual violence and encourage individuals to take responsibility for preventing it. The campaign includes educational programs on college campuses, public service announcements, and partnerships with advocacy organizations to foster a culture that actively challenges rape myths and promotes bystander intervention (Lee et al., 2023). Additionally, the Violence Against Women Act (VAWA), first enacted in 1994 and reauthorized multiple times, provides critical funding for victim services, legal assistance, and law enforcement training, reflecting a sustained national commitment to addressing sexual violence through legal and social reforms (Modi et al., 2014).

In India, NGOs like the Delhi-based Center for Social Research (CSR; CSR India, 2017) and the Mumbai-based YWCA are leading efforts to challenge harmful cultural norms and improve support for survivors. CSR’s initiatives include educational programs that address patriarchal attitudes and promote gender equality, while YWCA provides counseling and legal assistance to survivors of sexual violence. These organizations work to break the silence surrounding rape and support survivors through advocacy and community outreach (Semahegn et al., 2017). Additionally, the Indian government has enacted the Criminal Law (Amendment) Act, 2013, in response to the high-profile Delhi gang rape case, which introduced stricter penalties for sexual offenses and improved legal definitions of consent and sexual violence (Bandewar et al., 2018). These national and local efforts reflect a growing recognition of the need for comprehensive strategies to prevent rape and support survivors in diverse cultural and legal contexts, the summary of the study is described in Table-2.

## Conclusion and recommendations

Preventing and managing rape is complex and multifaceted. Overcoming these challenges requires addressing cultural issues and improving legal frameworks. Enhancing social control mechanisms is also essential, along with addressing barriers to implementing these mechanisms. Educational programs and public awareness campaigns play a vital role, as do improved community vigilance and support services for survivors. Promoting cultural change and adapting to new challenges presented by digital media are also crucial steps.

For legal frameworks, the “2013 Criminal Law (Amendment) Act,” or Nirbhaya Act, reformed India’s sexual violence laws following the 2012 Delhi gang rape. It expanded the definition of rape, introduced severe penalties like the death penalty for repeat offenders, and mandated fast-track courts for survivors. While the Act increased reporting, implementation challenges persisted. As an example of improving social norms, Nancy Glass from Johns Hopkins University discussed UNICEF’s Communities Care (CC) program, implemented with local and global partners in South Sudan and Somalia. The program aims to strengthen positive social norms that protect women and girls from violence and to transform those that condone or conceal gender-based violence (National Academies of Sciences, Engineering, and Medicine et al., 2018b). The “2018 Amendment” further strengthened penalties for crimes against minors, clarified consent issues, and addressed police and military cases. The Supreme Court also issued guidelines to protect victims and streamline judicial processes, enhancing ongoing legal reforms in India (National Academies of Sciences, Engineering, and Medicine et al., 2018b). Additionally, Emerging communication technologies, such as social networking and mobile phones, are vital for young people’s socialization but also heighten the risk of sexual violence by increasing access to potential victims. These technologies facilitate sexually violent acts before, during, and after incidents. They create a false sense of connection prior to an assault, allow offenders to record non-consensual acts and threaten victims with distribution during the event, and enable harmful sharing and communication afterward. The unclear guidelines on acceptable online behaviors complicate responses, suggesting that while legal measures are necessary, promoting personal ethics and respect through primary prevention is crucial to addressing these issues (Philbrick et al., 2021). These are few examples, similarly, society can work towards a more comprehensive and effective strategy for preventing rape and ensuring justice for survivors.

**Table 1:** 

New perspective is added to literature:
This paper uniquely analyzes how emerging communication technologies facilitate sexual violence while emphasizing the need for a prevention approach focused on personal ethics and respect rather than solely legal frameworks. Focus on Multifaceted Prevention: The review emphasizes the interconnectedness of legal frameworks, educational programs, social control mechanisms, and cultural change in rape prevention. Global Burden of Sexual Violence: It highlights significant rates of underreporting influenced by cultural stigma and systemic barriers, which have been previously overlooked. Comprehensive Methodology: It utilizes a literature search across multiple databases, capturing a wide range of empirical findings from the last two decades. Community and Public Awareness: It shows the role of community initiatives and campaigns in reshaping societal attitudes towards consent and accountability. Coercive Dynamics: It addresses the impact of coercive relationship dynamics, particularly among youth, on perceptions of consent. Successful International Strategies: It showcases effective approaches from countries like Sweden (consent-based laws) and the UK (public awareness campaigns) as models for change. Coordination and Integration: It emphasizes the need for a coordinated international response that integrates legal reform with grassroots efforts to foster cultural shifts. Actionable Insights: It links empirical data with practical strategies, paving the way for more effective and sustainable interventions against sexual violence.
